# Identification of Salmonella enterica Serovar Typhi Genotypes by Use of Rapid Multiplex Ligation-Dependent Probe Amplification

**DOI:** 10.1128/JCM.01010-13

**Published:** 2013-09

**Authors:** Duy Pham Thanh, Nga Tran Vu Thieu, Chau Tran Thuy, Martin Lodén, Kiki Tuin, James I. Campbell, Nguyen Van Minh Hoang, Phat Voong Vinh, Jeremy J. Farrar, Kathryn E. Holt, Gordon Dougan, Stephen Baker

**Affiliations:** The Hospital for Tropical Diseases, Wellcome Trust Major Overseas Programme, Oxford University Clinical Research Unit, Ho Chi Minh City, Vietnama; Department of Microbiology and Immunology, The University of Melbourne, Melbourne, Australiab; MRC-Holland, Amsterdam, The Netherlandsc; The Wellcome Trust Sanger Institute, Hinxton, Cambridge, United Kingdomd; Centre for Tropical Diseases, University of Oxford, Oxford, United Kingdome; The London School of Hygiene and Tropical Medicine, London, United Kingdomf

## Abstract

Salmonella enterica serovar Typhi, the causative agent of typhoid fever, is highly clonal and genetically conserved, making isolate subtyping difficult. We describe a standardized multiplex ligation-dependent probe amplification (MLPA) genotyping scheme targeting 11 key phylogenetic markers of the *S*. Typhi genome. The MLPA method demonstrated 90% concordance with single nucleotide polymorphism (SNP) typing, the gold standard for *S*. Typhi genotyping, and had the ability to identify isolates of the H58 haplotype, which is associated with resistance to multiple antimicrobials. Additionally, the assay permitted the detection of fluoroquinolone resistance-associated mutations in the DNA gyrase-encoding gene *gyrA* and the topoisomerase gene *parC* with a sensitivity of 100%. The MLPA methodology is simple and reliable, providing phylogenetically and phenotypically relevant genotyping information. This MLPA scheme offers a more-sensitive and interpretable alternative to the nonphylogenetic subgrouping methodologies that are currently used in reference and research laboratories in areas where typhoid is endemic.

## INTRODUCTION

Typhoid fever, caused by Salmonella enterica serovar Typhi (*S*. Typhi), remains a significant cause of mortality and morbidity in many regions of the world (1, 2). *S*. Typhi is a pathogen that is exclusive to humans, and this exquisite level of host restriction is reflected in the genome and population structure of the organism (3). *S*. Typhi isolates share an exceptionally high level of genetic conservation, making informative subtyping for local and international epidemiology challenging (3). Many methods involving both phenotypic and genotypic techniques have been developed to differentiate between *S*. Typhi isolates. Phage typing was long considered the standard method for strain subtyping; however, this technique provides little or no phylogenetic information, is technically intensive, and is only performed in specialized reference laboratories (4, 5). Multilocus sequence typing (MLST) cannot productively be applied to *S*. Typhi because of its monophyletic nature (6). Other molecular techniques, including random amplified polymorphic DNA PCR (RAPD-PCR), pulsed-field gel electrophoresis (PFGE), and amplified fragment length polymorphisms (AFLP), have some discriminatory power but provide a limited ability to define absolute phylogenetic and epidemiological relationships between isolates (7–9). More recently, genome sequencing and the identification of single nucleotide polymorphisms (SNPs) have provided the first well-defined phylogenetic framework within which to assign *S*. Typhi isolates to irrefutable genotypes that can describe the population (10–13). These studies revealed that *S*. Typhi genotypes are globally dispersed but are increasingly dominated by a single genotype (H58), which commonly displays resistance to multiple antimicrobials. H58 isolates frequently harbor multidrug-resistant incompatibility group HI1 (IncHI1) plasmids, as well as mutations in the *gyrA* gene, which confer reduced susceptibility to fluoroquinolones (7, 13).

While SNP detection and genome sequencing are the gold standards for *S*. Typhi subtyping (7, 10, 11), these methodologies remain in the domain of research institutions, which have the facilities, expertise, and infrastructure to support these activities. Therefore, there is a translation gap that stands in the way of utilizing genomic information for subtyping *S*. Typhi in endemic locations. To address this, we have developed a simple transportable method for genotyping *S*. Typhi that can be performed in a laboratory with only modest molecular biology capabilities.

*S*. Typhi genomes harbor several genomic insertions and deletions (indels) that we hypothesize to be phylogenetically informative and may be used as markers for inferring genotypes within the *S*. Typhi population (12). Therefore, we predicted that by assaying for selected indels in the *S*. Typhi genome, we can infer genotype, rather than having to perform genome-wide SNP analysis or genome sequencing. Multiplex ligation-dependent probe amplification (MLPA) is a PCR method that permits multiple DNA sequence targets to be amplified with a single primer pair (14). The ability of the method to detect DNA signatures, as well as individual SNPs, increases its potential utility and provides a system that can be globally standardized. We have designed, validated, and used an MLPA assay to genotype *S*. Typhi isolates and investigate the molecular epidemiology of typhoid in Asia. Our data show that *S*. Typhi MLPA is simple, robust, and comparable to the gold standard of SNP typing, permitting the identification of the major genotypes, as well as mutations that are associated with reduced susceptibility to fluoroquinolones. The method presented here can be applied to other organisms and represents an alternative to MLST, PFGE, genome sequencing, and serotyping when the ability to perform these methods or the methods themselves are limited.

## MATERIALS AND METHODS

### Bacterial isolates.

A total of 227 Salmonella isolates (217 *S*. Typhi and 10 S. enterica serovar Paratyphi A) were used in this study (see Table S1 in the supplemental material). All bacterial isolates originated from previous investigations conducted in southern Vietnam between 1993 and 2005 or across seven other Asian countries between 2002 and 2004, as previously described (15). Nineteen *S*. Typhi isolates that were previously whole-genome sequenced were used as controls for SNP typing and validation of the MLPA method; these isolates were provided by the Wellcome Trust Sanger Institute (United Kingdom) collection and are additionally described in Table S1 in the supplemental material (12). The 10 *S*. Paratyphi A isolates originated from India and were included as an out-group for SNP genotyping and MLPA analysis.

### Microbiological methods.

All bacterial isolates were identified by standard biochemical tests and agglutination with Salmonella-specific antiserum (Murex Diagnostics, Dartford, United Kingdom). Antimicrobial susceptibilities were tested at the time of isolation by the modified Bauer-Kirby disk diffusion method, as recommended by the CLSI guidelines (16). The antimicrobial disks tested were chloramphenicol (CHL) (30 μg), ampicillin (AMP) (10 μg), trimethoprim-sulfamethoxazole (SXT) (1.25/23.75 μg), ofloxacin (OFX) (5 μg), ciprofloxacin (CIP) (5 μg), and nalidixic acid (NAL) (30 μg). MICs were determined by Etest against CIP, NAL, and OFX, according to the manufacturer's recommendations (AB Biodisk, Sweden). Mueller-Hinton agar and antimicrobial disks were purchased from UniPath, Basingstoke, United Kingdom. Escherichia coli strain ATCC 25922 and Staphylococcus aureus strain ATCC 25923 were used as control strains for these assays. The results were interpreted according to current CLSI guidelines (16). An isolate was defined as multidrug resistant (MDR) if it was resistant to chloramphenicol, trimethoprim-sulfamethoxazole, and ampicillin.

### PCR amplification and DNA sequencing.

DNA was extracted from all *S*. Typhi isolates using the Wizard genomic DNA extraction kit (Promega, Fitchburg, WI). Oligonucleotide primers for the amplification of the quinolone resistance-determining regions (QRDRs) in the *gyrA* and *parC* genes in *S*. Typhi were as described in Table 1. PCRs were performed under the following conditions: 92°C for 45 s, 55°C or 62°C (depending on the primer pair) for 45 s, and 74°C for 1 min, for 30 cycles, followed by a final extension step at 74°C for 2 min. DNA sequencing reactions were performed using the CEQ Dye Terminator cycle sequencing (DTCS) quick start kit (Beckman Coulter) and were sequenced using a CEQ 8000 capillary sequencer (Beckman Coulter). All resulting DNA sequences were analyzed using the CEQuence investigator CEQ2000XL (Beckman Coulter) before verification, alignment, and editing in BioEdit software (http://www.mbio.ncsu.edu/BioEdit/bioedit. html). BLASTn at the NCBI was used to compare all resulting *gyrA* and *parC* sequences to those of global *S*. Typhi isolates. PCR amplification was used to confirm the insertion or deletion of the target sequence across all *S*. Typhi isolates prior to probe design and MLPA analysis; the primers used are shown in Table 1. PCRs were performed using the following conditions: 92°C for 45 s, 55 to 62°C for 45 s, and 74°C for 45 s, for 30 cycles, followed by a final extension step at 74°C for 2 min (Table 1).

**Table 1:** Conventional PCR primers targeting *S*. Typhi indels and SNPs

Primer	Primer sequence (5′ to 3′)	Target^*a*^	Annealing temp (°C)	Amplification size (bp)
indelA_F	AGCGATGTGATGATCAGGATT	Indel A	55	418
indelA_R	AATGGCGTGTTCAGTGGATT			
indelB_F	TCCGTCTCTTTCTCCAGC	Indel B	55	368
indelB_R	AATTGATGCTGCTGCTGGACG			
indelC_F	ACGGGTGAAATACTCGAACG	Indel C	58	783
indelC_R	CACCAAGCAGATTGTTCAGG			
indelD_F	CGCTATTTTTTCCGCCCATGC	Indel D	60	1,451
indelD_R	TAATAACATCGGCGTGCCG			
indelE_F	CCGTCGCCAAAGTGACGC	Indel E	55	993
indelE_R	CCGTTGAATCGGAAGTAATAATCG			
indelF_F	AAGCAAATGCTTAGCACCAC	Indel F	55	180
indelF_R	CAATGCATAAAGTTAATTTAATCAGGA			
indelK_F	ATGGGTGAGCGCCTCTTTGG	Indel K	55	155
indelK_R	GACTGGCTGGACATTTTGTG			
indelN_F	ATGTTTCATGTGTGGGTAGGGTTGCC	Indel N	58	720
indelN_R	AAGAATGCCCATTGAGCGG			
indelQ_F	CAACACCCGTGCGGACGAT	Indel Q	60	841
indelQ_R	AGCTTACTTCCGGCTCCGAC			
indelS_F	TTGGTGATAAAATTGGCTCGGG	Indel S	60	1,116
indelS_R	AAAGAATGGAAACCAGAGTTTCC			
indelH_F	GTGGCAAAACAACGCATCG	Indel H	62	6,560
indelH_R	CGGTGGAGTTAGTGATGCTGA			
GYRA/P1	TGTCCGAGATGGCCTGAAGC	*gyrA*	55	347
GYRA/P2	TACCGTCATAAGTTATCCACG			
StmparC1	CTATGCGATGTCAGAGCTGG	*parC*	62	270
StmparC2	TAACAGCAGCTCGGCGTATT			

a Indel sites correspond to those shown in Fig. 1.

### MLPA.

All MLPA reagents were manufactured and supplied by MRC-Holland (Amsterdam, The Netherlands). Sixteen *S*. Typhi-specific MLPA probes were designed according to the manufacturer's recommendations. The probes are described in Table 2 and target 11 *S*. Typhi indels (B, Q, A, K, E, F, S, C, H, N, and D) (Fig. 1). MLPA was performed according to a standard protocol developed by MRC-Holland, as previously described (14). Briefly, *S*. Typhi genomic DNA was diluted with Tris-EDTA (TE) buffer to final volume of 5 μl (50 to 200 ng) and heated at 98°C for 10 min in a thermocycler (Bio-Rad). After denaturation, the DNA was hybridized with MLPA reagents at 60°C. After overnight incubation, ligation was performed at 54°C for 15 min, followed by 98°C for 5 min. Ten microliters of the ligation mixture was added to 30 μl of PCR buffer. After equilibration at 60°C, 10 μl of SALSA PCR master mix, containing the labeled forward primer 5′-FAM-GGGTTCCCTAAGGGTTGGA-3′ (FAM, 6-carboxylfluorescein) and the unlabeled reverse primer 5′-GTGCCAGCAAGATCCAATCTAGA-3′, was added. PCR amplification was performed using 35 cycles of 95°C for 30 s, 60°C for 30 s, and 72°C for 45 s, followed by 72°C for 20 min. The resulting MLPA amplicons were examined by agarose gel (3%) electrophoresis and by fragment analysis using an ABI 3130xl capillary electrophoresis system (Applied Biosystems). For fragment analysis, 0.5 μl of PCR amplicon was mixed with 9.25 μl of Hi-Di formamide and 0.25 μl 500 LIZ size standard (GeneScan; Applied Biosystems). The mixture was incubated for 3 min at 95°C, chilled for 10 min, and analyzed. The resulting fragment analysis data were analyzed using GeneMapper v4.0 (Applied Biosystems). Data were analyzed using BioNumerics (Applied Maths, Belgium), and phylogenetic trees were drawn using Dendroscope v2.3.

**Table 2:** MLPA probe targets and sequences

MLPA target	Fragment/amplicon length (bp)	MLPA probe sequence^*a*^
*parC*^*b*^	130	TCAATACGACTCTAGATTAGACACCATGGCTTCATAGCAGGCG**A**
		TGTCGCCATGCGGGTGATACTTACCTATTATGCTGAGC
Indel B	139	TTCCGGTCAGACTTGGACGCAAAATACAGGTA
		CGAAAGGCCAGCTCGTGAAAATCCATAGAGGGGCT
Indel Q	158	TTATGGACGCTGGTGCAGCAGGAG
		CCCTCCCATCCGTTAATTGTTAGTACCACGCTGAACACCG
Indel A	175	TTTTAGTCCATCACGCGCTTCAACATCCGTTC
		TATCAGGATGTTGAAAAAGTGGTGCGCATTAATCCACTGAACAC
Indel K	204	TCGACTGGCTGGACATTTTGTGGTTC
		TCTGCCGATGCGCTGGAGCAGCTTTGTGATGCGCT
*gyrA^b,c^*	238	CCAGCGAGAATGGCTGCGCCATACGAACGA
		**A**ATCGCCGTGGGGATGGTATTTACCGATTACGTCACCAACGAC
Indel E	252	GGAACATGGCGATATCAAATCCATCAATG
		AAAGCGTCCAGTCCCTGTTTGATGCTCAGTCCCTG
*aroC*	267	GACAACAACGATAACGGAGCCGTGATGGCA
		GGAAACACAATTGGACAACTCTTTCGCGTAACCACTTTCG
Indel S	275	GCTCTCTGGCTTTTTGAACACAGAAATGG
		TCGAGTGAAATCAGCTTTGACTTAGCCCTTTTATAAAGCCTTGCGGC
Indel F	308	GCAGTCAGATTTGAACGTCCGGCATAA
		AAATAAGTGCTATTAAGCTCTTCAACTGTATCCATACTCTAATTTCCTG
Indel C	362	CTAAAGCGTCGTTGATAATGGTGTAACGGT
		GAACTGTCACAGGTCGTGGCGTTATCTTCGATAAACTTCACCT
*fliC*	374	CTGTTGACCCAGAATAACCTGAACAAATCC
		CAGTCCGCACTGGGCACTGCTATCGAGCGTTTGT
Indel H	414	CTTGCTTTACCGAGCAGCATATTCTTTCCGCT
		ATGACCGTTCAGTATTTGCAGAAGAAAGGCTTTCAGGTTCAGCC
*ssaV*	429	CCTGATTCCCTCACCTAACCATGAAC
		GCATTGCGACTCCAGAAATTTTATTTGTCGATGATGTAATCGTAACC
Indel N	465	CCAGCACAGGCGTATTATCGCTAACACGCT
		CACCAACATAGTCGATTTCATGCCAGTAAACTATTGGTAGCCTGGT
Indel D	494	CCTGGTCAAGCGTTAACTTTATTACTGCCCAT
		GACGGCTTCACGCTGAACGATCTGGTGTCTTATAACGA

a Probes comprise left and right hybridization sequences.

b Probe contains a nucleotide to detect a specific SNP in the QRDR region (boldface and underlined).

c *gyrA* probe requires a spanning oligonucleotide to detect mutations at codons 83 and 87 (TGGT**GTC**ATACACTGC**GA**).

**Fig 1:**
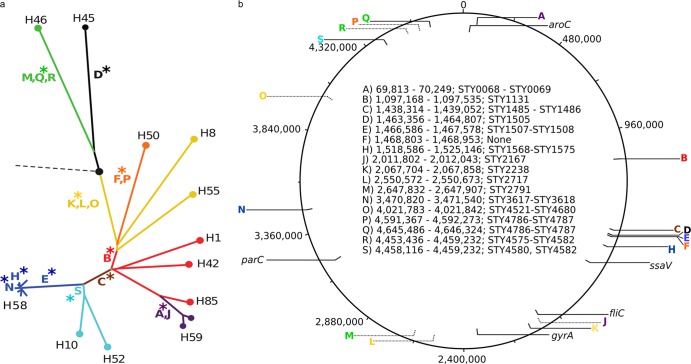
Indels in the Salmonella Typhi genome. (a) Phylogenetic tree of *S*. Typhi derived from SNP data, adapted from Holt et al. (12). The broken line with a black circle designates the ancestral root; all major haplotypes are highlighted (e.g., H58). Capitalized colored letters represent 17 different indels, the position of the indels on the tree indicates the branch with which they are associated, and the asterisks highlight the 11 indels selected for MLPA. (b) Genomic map of *S*. Typhi strain CT18 from 1 to 4,800,000 bp (coordinates shown). Highlighted are the genomic positions and the strand of the 17 indels and the *aroC*, *ssaV*, *fliC*, *gyrA*, and *parC* genes. In the center of the map is a description of the 17 indels (A to S), showing the genomic coordinates (with respect to *S*. Typhi CT18) and the affected coding sequence(s).

### Single nucleotide polymorphism genotyping.

A cross-sectional subset of 73 *S*. Typhi isolates was arbitrarily selected to encompass a range of locations, antimicrobial resistance patterns, studies in Vietnam, and dates of isolation for gold-standard SNP genotyping using the Illumina GoldenGate platform (see Table S1 in the supplemental material). DNA was extracted from all *S*. Typhi isolates as described above, and concentrations were assessed using the Quant-iT kit (Invitrogen, Carlsbad, CA) prior to SNP typing. The chromosomal haplotype of *S*. Typhi isolates was determined based on the SNPs present at 1,485 chromosomal loci identified previously from genome-wide surveys (12, 17). All loci were interrogated using a GoldenGate (Illumina) custom assay according to the manufacturer's standard protocols, as described previously (10, 11, 18, 19). SNP calls were generated from raw fluorescence signal data by clustering with a modified version of Illuminus, as described previously (10, 11, 18, 19). SNP alleles were concatenated to generate a multiple-sequence alignment; maximum likelihood phylogenetic trees were fitted to each alignment using RAxML with a general time reversible plus gamma (GTR+γ) model and 1,000 bootstraps as previously described (11, 20).

## RESULTS

### Development of MLPA to detect phylogenetically informative indels in the *S*. Typhi genome.

Data gleaned from genome sequencing predicted that the presence or absence of certain indels within the *S*. Typhi chromosome might be phylogenetically informative markers for assigning *S*. Typhi isolates to the genotype groups originally defined by SNP analysis (12). Consequently, conventional PCR assays were designed to independently detect 11 of these previously described indels (12). Each target (A, B, C, D, E, F, H, K, N, Q, and S) was selected to represent one of the main branches of the *S*. Typhi phylogenetic tree, and where two indels were present on one branch, one was selected to avoid redundancy (Table 1, Fig. 1).

Conventional PCR amplification, detecting indels at the 11 target loci, was performed on DNA extracted from the 19 *S*. Typhi isolates that had been whole-genome sequenced (12) (Table 3). The PCR amplification results (PCR^+^/indel^+^ and PCR^−^/indel^−^) corresponded precisely with the genome sequencing data, confirming the phylogenetic potential of these 11 indel targets in the selected isolates.

**Table 3:** Comparison of *S*. Typhi MLPA indel genotyping and genome sequencing

*S*. Typhi isolate^*a*^	Location of isolation	Haplotype^*a*^	Predicted indel pattern as determined by:	
			Sequencing^*a*^	MLPA
E98-2068	Bangladesh	H42	KB	KB
CT18	Vietnam	H1	KB	KB
J185SM	Indonesia	H85	KB	KB
404ty	Indonesia	H59	KB	KB
E03-4983	Indonesia	H59	KBA	KBA
AG3	Vietnam	H58	KBCE	KBCE
8(04)-N	Vietnam	H58	KBCE	KBCE
E02-2759	India	H58	KBCE	KBCE
E03-9804	India	H58	KBCE	KBCE
ISP-03-07467	Morocco	H58	KBCE	KBCE
ISP-04-06979	Central Africa	H58	KBCE	KBCE
150(98)S	Vietnam	H63	KBCES	KBCES
E98-0664	Kenya	H55	K	K
M223	Unknown	H8	K	K
Ty2	Russia	H10	KBCS	KBCS
E01-6750	Senegal	H52	KBCS	KBCS
E98-3139	Mexico	H50	KF	KF
E00-7866	Morocco	H46	Q	Q
E02-1180	India	H45	D	D

a See Holt et al. (12).

**Fig 2:**
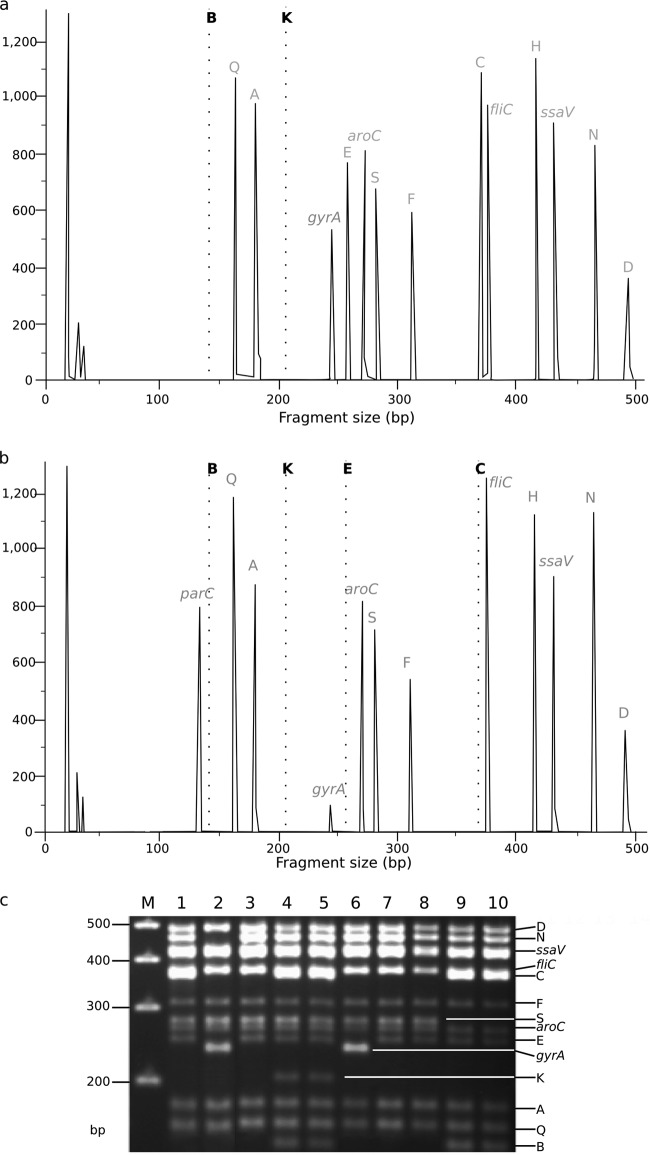
Salmonella Typhi MLPA indel typing. (a) Fragment analysis generated by MLPA of *S*. Typhi isolate 113 from Vietnam, demonstrating a KB (H1) indel pattern *S*. Typhi by MLPA (KB peaks are missing, indicated by broken lines). Each peak corresponds with the fluorescence intensity of amplicons targeting specific loci (labeled at the top of each peak), including a large peak at 238 bp associated with a single *gyrA* mutation at codon 83. (b) Fragment analysis generated by MLPA of *S*. Typhi isolate 9019 from Vietnam, demonstrating a KBCE (H58) indel pattern by MLPA (KBCE peaks are missing, indicated by broken lines). This isolate has two *gyrA* mutations at codons 83 and 87 and a *parC* mutation, as determined by a diminished peak at 238 bp and large peak at 130 bp, respectively. (c) Representative fragment analysis of *S*. Typhi MLPA amplicons by agarose gel electrophoresis. Fourteen bands are visible and correspond with the predicted MLPA amplicon lengths shown in Table 2. Lane M, 100-bp marker; lane 1, *S*. Typhi 113/Vietnam (panel a) KB (*gyrA* mutation^−^); lane 2, *S*. Typhi A763/India-KBCEN (*gyrA* mutation^+^); lane 3, *S*. Typhi TY8339/Vietnam-KBC (*gyrA* mutation^−^); lane 4, *S*. Typhi DOMIC14/China-ancestral (*gyrA* mutation^−^); lane 5, *S*. Typhi DOMIC43/China-ancestral (*gyrA* mutation^−^); lane 6, *S*. Typhi TY7045/Vietnam-KBCE (*gyrA* mutation^+^); lane 7, *S*. Typhi J348BM/Indonesia-KBC (*gyrA* mutation^−^); lane 8, *S*. Typhi AS3357/Bangladesh-KBC (*gyrA* mutation^−^); lane 9, *S*. Typhi DOMI40/China-KS (*gyrA* mutation^−^); lane 10, DOMI38/China-KS (*gyrA* mutation^−^).

An MLPA probe set was designed to detect the same 11 indels, as well as SNPs in the *gyrA* and *parC* genes and sequences within the conserved *S*. Typhi genes *aroC*, *ssaV*, and *fliC* to serve as controls for hybridization, amplification, and detection. The probe sequences and the predicted amplicon length for each target are shown in Table 2. The *gyrA* and *parC* probes were designed to detect SNPs associated with reduced susceptibility to fluoroquinolones within the QRDRs. Although several mutations have been identified at *gyrA* codon 83, the most common mutation associated with fluoroquinolone resistance (TCC to TTC) (15, 21) changes serine to phenylalanine (S83F); thus, the *gyrA* probe was designed to target this mutation. Additionally, a spanning probe was added to differentiate mutations at codons 83 and 87 within *gyrA*; it was designed so that a wild-type allele would not yield a detectable DNA fragment and so other (non-S83F) mutations at codons 83 and 87 would generate a product of decreased intensity in comparison to any product yielded from the S83F mutation. The *parC* probe was designed to detect the most common fluoroquinolone resistance mutation (AGC to ATC), which changes serine to isoleucine at codon 80, and to yield a product only when an *S*. Typhi isolate contained the S80I mutation (15, 21). The indel type was inferred by the absence of amplification or of peaks following fragment analysis (Fig. 2).

### Validation of MLPA for *S*. Typhi genotyping and detection of *gyrA* and *parC* mutations.

MLPA was performed using DNA prepared from 19 global *S*. Typhi isolates that were previously whole-genome sequenced (Table 3) (12). Analysis of the DNA fragments generated from these PCR amplifications demonstrated that all probes, including the controls, performed as anticipated, as they produced measurable and reproducible amplicons of the predicted sizes. Representative examples of the resulting DNA fragment analysis and gel electrophoresis are shown in Fig. 2. Notably, the probes targeting the *gyrA* and *parC* mutations were able to distinguish between isolates with and without mutations in the QRDR. Figure 2 shows that the *parC* amplicon was detectable in isolates with a *parC* mutation, and a diminished *gyrA* signal was visible in isolates with a mutation at codons 83 and 87. The indel and QRDR patterns of the *S*. Typhi isolates yielded by MLPA corresponded precisely with the indel patterns and the *gyrA* and *parC* mutations anticipated from both the genome sequences and conventional PCR (Table 3).

**Fig 3:**
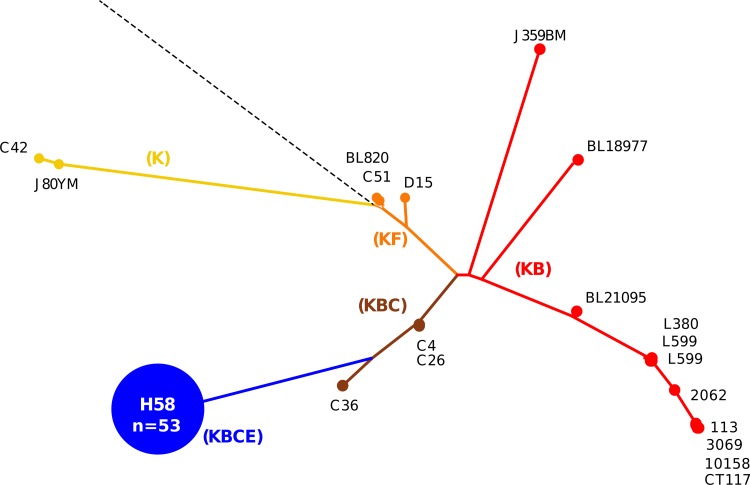
Comparison of MLPA and SNP genotyping for *S*. Typhi. The phylogenetic tree was determined by detecting SNP variations at 1,485 previously identified loci across the *S*. Typhi genomes of 73 isolates using a custom GoldenGate assay (see Table S1 in the supplemental material). The resulting tree was used to infer indel patterns (colored capitalized letters) and their associated branches. Examples of *S*. Typhi strains at pivotal locations throughout the tree are named, and the H58 group (KBCE indel pattern), consisting of 54 strains, is highlighted in blue.

Next, 73 *S*. Typhi isolates of undetermined genotype from Bangladesh, China, India, Indonesia, Laos, Pakistan, and Vietnam and 10 *S*. Paratyphi A isolates from India (as a control) were typed by detecting SNP variations at 1,485 previously identified loci across the *S*. Typhi genome using a custom GoldenGate assay (see Table S2 in the supplemental material). These data were used to determine a phylogenetic tree, which subdivided the 73 *S*. Typhi isolates into six main genotype groups, broadly corresponding to those previously defined as H1, H42, H50, H52, H58, and H59 (Fig. 1 and 3) (3, 12). Approximately three-quarters (54/73) of the genotyped *S*. Typhi isolates belonged to the H58 genotype, including isolates originating from Bangladesh, India, Laos, Pakistan, and Vietnam. As predicted, the 10 *S*. Paratyphi A isolates fell into the ancestral group of *S*. Typhi via SNP typing, i.e., were ancestral for all SNPs, as all mutations were *S*. Typhi specific (data not shown).

MLPA was then performed on the same 73 *S*. Typhi isolates (Fig. 3). The H58 complex has a predicted indel pattern of KBCE and all 54 of the H58 (as determined by SNP typing) *S*. Typhi isolates produced this pattern (Fig. 3). The MLPA indel patterns were also consistent with the SNP genotypes in all of the H1 (KB), H42 (KB), and H59 (KB) isolates (9/9 total). The five isolates belonging to H52 (predicted indel pattern KBCS) produced 3 KBC, 1 KBCS, and 1 KBCD MLPA pattern. The five H50 *S*. Typhi isolates (with a predicted KF indel pattern) produced four K patterns and one KS pattern. Thus, genotype predictions based on the detection of MLPA indel patterns matched those predicted from SNP-based genotype assignments in 66/73 (90.4%) of the *S*. Typhi isolates. The 10 *S*. Paratyphi A isolates produced amplicons for the *aroC*, *fliC*, and *ssaV* genes and produced peaks corresponding with an indel pattern of SF, which does not correlate with any predicted *S*. Typhi genotype (see Table S1 in the supplemental material).

### MLPA of *S*. Typhi from eight Asian countries.

**Fig 4:**
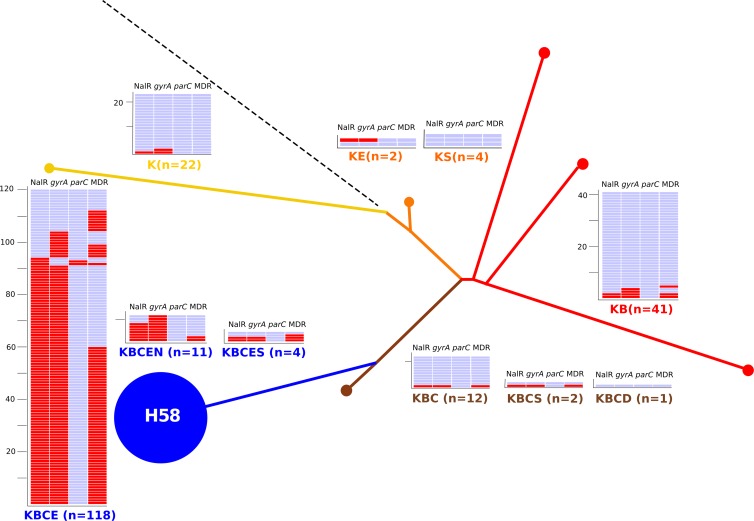
MLPA indel typing of 217 Asian *S*. Typhi isolates. The phylogenetic tree was determined by detecting SNP variations at 1,485 previously identified loci across the *S*. Typhi genomes of 73 isolates using a custom GoldenGate assay inferring the genotype of 217 Asian *S*. Typhi isolates by MLPA. The color coding and labeling is as shown in Fig. 3. The numbers of isolates with a specific indel type are shown, color coded, and associated with a heat map showing the presence (red) or absence (blue) of a NALR phenotype, a mutation in the QRDR of the *gyrA* gene by MLPA, a mutation in the QRDR of the *parC* gene by MLPA, and an MDR phenotype.

Subsequently, MLPA typing was performed on an additional 144 *S*. Typhi isolates of undetermined genotype from Vietnam (88 isolates) and seven other Asian countries. The MLPA results were combined with data from the 73 *S*. Typhi isolates typed during validation (see Tables S1 and S2 in the supplemental material). According to antimicrobial susceptibility testing, 119/217 (54.8%) isolates were nalidixic acid resistant (NALR) and 82/217 (37.8%) isolates were MDR (Table 4), and 71 isolates (32.7%) were both NALR and MDR (Fig. 4). We also used the *gyrA* and *parC* MLPA amplicons to detect the presence of NALR-conferring mutations and compared these data to the antimicrobial susceptibility results (Table 4, Fig. 4).

**Table 4:** MLPA indel genotyping of 217 Asian *S*. Typhi isolates

MLPA indel pattern^*a*^	No. of isolates	Country of isolation (no.)	No. with *gyrA* mutation detected by MLPA	No. with resistance type	
				Nalidixic acid	Multidrug
KBCE	118	Vietnam (79), Bangladesh (9), India (18), Pakistan (5), Laos (6), Nepal (1)	93	99	73
KBCEN	10	India (5), Nepal (3), Pakistan (2)	7	10	2
KBCES	4	Vietnam (4)	2	2	3
KB	41	Vietnam (4), Bangladesh (5), India (3), Pakistan (5), Laos (8), Nepal (8), China (1), Indonesia (7)	3	5	4
KBC	12	Vietnam (1), Bangladesh (1) China (9), Indonesia (1)	0	0	0
KBCS	2	Laos (2)	0	0	0
KBCD	1	Laos (1)	0	0	0
K	22	Bangladesh (1), India (2), Pakistan (6), Nepal (2), China (1), Indonesia (6), Laos (4)	1	2	0
KS	3	China (3)	0	0	0
KE	2	India (1), Bangladesh (1)	0	1	0
No deletions (ancestral)	2	China (2)	0	0	0
Total isolates	217		106	119	82

a Five *S*. Paratyphi controls gave an indel pattern of SF and were not included in this table (see Table S1 in the supplemental material).

We detected 10 different indel patterns in the 217 *S*. Typhi isolates (Fig. 4, Table 4). Fifty-nine percent of the isolates (128/217) produced a KBCE or KBCEN MLPA pattern, corresponding to the H58 genotype (Table 4, Fig. 4). This H58 group included 90% of isolates from Vietnam (79/88), 79% of isolates from India (23/29), 53% of isolates from Bangladesh (9/17), 39% of isolates from Pakistan (7/18), 31% of isolates from Laos (6/21), and 29% of isolates from Nepal (4/14). We additionally identified 4 isolates with a KBCES indel pattern, and 41 isolates (18%) with a KB indel pattern (H42 haplotype) were identified and spanned all eight Asian countries. Twelve isolates (6%) produced a KBC (H52) indel pattern, and 22/217 (10%) isolates produced a K indel pattern. An additional three indel patterns (KE, KS, and KBCS) were identified across seven isolates, and two Chinese isolates produced amplicons for all loci and were deemed to be ancestral.

MLPA detected 106 isolates with an amplicon consistent with a mutation in *gyrA* and two isolates with a mutation in *parC* and a double mutation in *gyrA*; all were confirmed by sequencing (Fig. 4, Table 4). Of the 119 isolates with phenotypic NALR, 106 (90%) tested positive for a *gyrA* mutation by MLPA (Table 4). The MLPA analysis revealed that the H58 indel patterns KBCE and KBCEN had an increased proportion of NALR isolates compared to other indel patterns (H58 NALR, non-H58 NALR) (*P* < 0.0001, Fisher's exact test; see Table 4). Furthermore, the same H58 indel group had an increased proportion of MDR strains compared to other indel patterns (H58 MDR versus non-H58 MDR) (*P* < 0.0001, Fisher's exact test).

## DISCUSSION

Phylogenetically informative subgrouping is vital for understanding the local and regional strain circulations of bacterial pathogens. Diagnostic, research, and reference laboratories use a range of typing methods for recording and reporting bacterial diversity with a variety of pathogens (22, 23). However, these methods often lack reproducibility, sensitivity, and phylogenetic relevance (24). The consequence of these limitations is an inability to perform robust epidemiological investigations of circulating bacterial pathogens. Genomics offers a solution to these limitations, and DNA sequence-based methodologies can be used to improve bacterial subtyping (10, 25, 26). However, whole-genome sequencing is currently too specialized and expensive to be employed in areas where typhoid is endemic. We aimed to address this and developed a simple and reproducible *S*. Typhi subtyping method that can be performed in locations with limited molecular biology equipment. The MLPA method utilizes a limited number of steps and can be conducted with a standardized set of reagents and protocols. The method we developed performs well and can be made commercially available, depending on the demand within the enteric fever research community. It is difficult to perform a direct cost comparison of MLPA and sequencing, as demand impacts the cost of the MLPA kit; however, we estimate a cost in the realm of $10 to $20 per isolate for MLPA compared to the current cost of $500 to $1,000 per isolate for next-generation sequencing (price estimated on 5× to 15× coverage of a whole *S*. Typhi genome, using a 454 Junior next-generation sequencer with current costs in Vietnam; this could be reduced by using commercial sequencing facilities).

The MLPA method successfully detected all 11 phylogenetically informative indels, permitting us to infer a genotype for each. In our test set of 73 *S*. Typhi isolates, MLPA indel patterns accurately predicted SNP-based genotype assignments in 90% of the strains. For the remaining isolates, the MLPA profiles were a combination of indels that were similar, but not identical, to those we anticipated from SNP genotypes. Our method also permitted the incorporation of probes targeting the QRDR regions of *gyrA* and *parC*. The ability to detect mutations in *gyrA* and *parC* is highly relevant for epidemiological studies of *S*. Typhi, as NALR *S*. Typhi has been reported in the majority of countries where typhoid is endemic (15, 19, 21, 27). NALR is a reliable marker of isolates with a reduced susceptibility to fluoroquinolones, the most common group of antimicrobials used to treat typhoid. When treated with fluoroquinolones, NALR *S*. Typhi is associated with an extended fever clearance time, prolonged antimicrobial therapy, and an increased risk of treatment failure (28). Therefore, knowing the prevalence of *gyrA* mutations in endemic locations is important for treatment and is of value to epidemiologists, clinicians, and those making guidelines for clinical care.

Among the 106 isolates in which NALR mutations were detected by MLPA, all *gyrA* and *parC* mutations were confirmed by direct PCR amplicon sequencing, indicating 100% specificity for the detection of mutations in these genes using MLPA. Notably, we detected only two strains containing the *parC* mutation; these strains were from India, confirming our previous observation that this mutation is rare and has only been identified in strains circulating in India (15). There were 13 NALR isolates that tested negative for *gyrA* and *parC* mutations using MLPA; direct sequencing of the amplicons confirmed the MLPA data. We suggest that this 10% discrepancy between MLPA and NALR phenotypic data was caused by either fluctuation in zone size readings during antimicrobial susceptibility testing by disk diffusion or by novel mutations in the QRDR that were not represented in this assay (15). The successful use of MLPA to identify point mutations is encouraging and suggests that this method can be adapted to establish a simple and robust alternative to SNP typing for *S*. Typhi and other monomorphic pathogens, which may be more phylogenetically informative than the use of genomic indels.

The potential limitations of our work include the lack of an incHI1 plasmid probe, a bias toward isolates from Vietnam, and the fact that some indel profiles are shared by several genetically diverse *S*. Typhi genotypes, while other indel profiles define much more homogeneous groups. Hence, the resolution offered by indel typing is somewhat varied. We suggest that the discrepancies between SNP and MLPA typing are induced by the stability of some indels in different lineages, predicting that some indel loci may not be as phylogenetically informative as others. However, notwithstanding these limitations, our comparison of genome-wide SNP with MLPA typing showed that MLPA was able to distinguish between five common *S*. Typhi isolates and, most significantly, was able to correctly identify H58 strains from Vietnam and across Asia. Principally, we were able to distinguish H58 strains with and without *gyrA* mutations with 100% accuracy. It has been shown that H58 strains currently predominate in the majority of endemic locations, and there is an association between H58 strains and S83F *gyrA* mutations and an MDR phenotype (3, 13). The MLPA data confirmed that H58 *S*. Typhi is dominant in Vietnam and is widely dispersed across Asia, and it is associated with both NALR and MDR in these regions. As the use of high-resolution SNP typing and genome sequencing have previously reported similar associations, the ability of MLPA to reproduce this result confirms its reliability and potential utility for epidemiological investigations. We advocate a new system for the subtyping of *S*. Typhi and propose that indel detection, either by MLPA or conventional PCR, be adopted until SNP typing becomes more routine. We aim to develop a global Web-based *S*. Typhi, *S*. Paratyphi A, invasive nontyphoidal Salmonella reference network whereby serotyping is combined with simple genotyping information, antimicrobial resistance profiling, and the time and date of isolation. The use of a standardized kit-based assay, such as the one presented here, that can be adapted through targeted genome sequencing would allow for routine data sharing and a global resource for assessing international epidemiology and defining appropriate measures for therapy and vaccination.

In conclusion, we have developed a simple, reliable, and epidemiologically relevant method that can be used in laboratories with modest molecular biology equipment to subgroup *S*. Typhi and identify common NALR-associated mutations. This MLPA method can distinguish between strains that currently dominate the global population structure of *S*. Typhi, offering a more sensitive and simple alternative to other subgrouping methodologies that are currently being used in low- and middle-income countries.

## Supplementary Material

Supplemental material
