# Supplementary material for: Identification of Salmonella enterica Serovar Typhi Genotypes by Use of Rapid Multiplex Ligation-Dependent Probe Amplification
Source: J Clin Microbiol. 2013 Sep;51(9):2950–8. doi: 10.1128/JCM.01010-13 (PMC3754622; doi:10.1128/JCM.01010-13)
Supplement: Supplemental material [file supp_51_9_2950__index.html]

Identification of Salmonella enterica Serovar Typhi Genotypes by Use of Rapid Multiplex Ligation-Dependent Probe Amplification — Supplemental material 

# Identification of Salmonella enterica Serovar Typhi Genotypes by Use of Rapid Multiplex Ligation-Dependent Probe Amplification

## Supplemental material

**Files in this Data Supplement:**

- Supplemental file 1 -

  Table S1 (Bacterial isolates used in this study)

  XLSX, 71K
- Supplemental file 2 -

  Table S2 (Comparison of MLPA and SNP genotyping)

  XLSX, 52K
